# Evaluation of Nutritional Values of Edible Algal Species Using a Shortwave Infrared Hyperspectral Imaging and Machine Learning Technique

**DOI:** 10.3390/foods13142277

**Published:** 2024-07-19

**Authors:** Tiziana Amoriello, Francesco Mellara, Monica Amoriello, Roberto Ciccoritti

**Affiliations:** 1 CREA Research Centre for Food and Nutrition, Via Ardeatina 546, 00178 Rome, Italy; francesco.mellara@crea.gov.it; 2 CREA Central Administration, Via Archimede 59, 00197 Rome, Italy; monica.amoriello@crea.gov.it; 3 CREA Research Centre for Olive, Citrus and Tree Fruit, Via di Fioranello 52, 00134 Rome, Italy

**Keywords:** protein, lipid, fiber, microalgae, seaweeds

## Abstract

In recent years, the growing demand for algae in Western countries is due to their richness in nutrients and bioactive compounds, and their use as ingredients for foods, cosmetics, nutraceuticals, fertilizers, biofuels,, etc. Evaluation of the qualitative characteristics of algae involves assessing their physicochemical and nutritional components to determine their suitability for specific end uses, but this assessment is generally performed using destructive, expensive, and time-consuming traditional chemical analyses, and requires sample preparation. The hyperspectral imaging (HSI) technique has been successfully applied in food quality assessment and control and has the potential to overcome the limitations of traditional biochemical methods. In this study, the nutritional profile (proteins, lipids, and fibers) of seventeen edible macro- and microalgae species widely grown throughout the world were investigated using traditional methods. Moreover, a shortwave infrared (SWIR) hyperspectral imaging device and artificial neural network (ANN) algorithms were used to develop multi-species models for proteins, lipids, and fibers. The predictive power of the models was characterized by different metrics, which showed very high predictive performances for all nutritional parameters (for example, R^2^ = 0.9952, 0.9767, 0.9828 for proteins, lipids, and fibers, respectively). Our results demonstrated the ability of SWIR hyperspectral imaging coupled with ANN algorithms in quantifying biomolecules in algal species in a fast and sustainable way.

## 1. Introduction

Algae are a broad group of photosynthetic organisms of different dimensions, shapes, and colors, and with different filament complexities, from simple to branched [1]. They are widely spread across all of the world’s biogeographic areas, having a robust ability to adapt to different environmental conditions (temperature, light, nutrient concentration, hydro dynamism, etc.) [2]. Algae are classified as red (Rhodophyta), brown (Phaeophyceae), green (Chlorophyta), and blue-green (Cyanophyta) algae depending on the nature of their pigments. The first three phyla are often called seaweeds or macroalgae. They are macroscopic marine algae, whose length can reach tens of meters [3]. Actually, there are around 7533 red, 2133 brown and 8191 green species in nature [4]. Microalgae are microscopic organisms naturally found in marine environments or fresh water. Although it is estimated that there are many microalgal species, approximately 44,000 have currently been studied [5]. Among these, only a small number, such as *Auxenochlorella vulgaris* and *Limnospira platensis*, are commercially relevant [5].

According to the FAO, in 2021 the production of algae was 36 million tonnes (wet weight), mainly from aquaculture and marine aquaculture [6]. The top producers were China, Indonesia, the Republic of Korea, and the Philippines (with shares of 60%, 25%, 5%, and 4%, respectively). The majority of algae (more than 95 percent, dominated by seaweeds) is harvested from the sea. The major exporters of algae were the Republic of Korea, Indonesia, and China, while China, Japan, and the USA were the leading importers. Algae are consumed on a large scale in Far Eastern countries (especially in Japan, China, Korea, the Philippines, and Indonesia), Mexico, Africa, and, on a smaller scale, in Europe. However, their use has greatly increased in recent years even in Western countries. According to Meticulous Research^®^ (www.meticulousresearch.com/product/algae-market-5424 accessed on 10 July 2024), the algae market is expected to reach USD 29.8 billion by 2030, from USD 22.1 billion in 2024 [7]. This positive trend can be attributed to the growing popularity of algae due to their sustainability and environmental and nutritional benefits. In fact, algae can be used as human foods, cosmetics, nutraceuticals, pharmaceuticals, plastics, fertilizers, biofuels, and for the extraction of industrial gums (phytocolloids and gelling agents, such as agar and carrageenan from Rhodophyta and alginates from Phaeophyceae) and chemicals [1,8,9].

Because the world population is expected to increase up to 9.7 billion people by 2050, and to ensure nutritious, adequate, and safe food for all, it is necessary to identify new and sustainable sources of food. From this point of view, algae can play a promising role in global food security. In fact, increasing attention has been directed at the use of both microalgae and seaweeds for the development of functional foods, such as pasta, bread, biscuits, snacks, vegetable soups, noodles, stews, burgers, garnishes, chips, candy bars or gums, yogurts, ice creams, drinks, etc., due to the great variety of nutrients essential for human health that they possess [4,8,10,11]. Algae are increasingly recognized as nutritious and healthy foods that are claimed to be excellent sources of proteins, lipids, carbohydrates, dietary fibers, vitamins (A; B-complex, including B12; C; D; E; pantothenic acid; and folic acid), minerals (calcium, magnesium, potassium, iodine, sodium, phosphorus, nickel, chromium, selenium, iron, zinc, and manganese), pigments, and other biological compounds, such as carotenoids, polyphenols, antioxidants, and phytoestrogens,, etc. [1,12,13]. Due to all these properties, the World Health Organization (WHO) has recommended algae as an option to remedy malnutrition, as reported by Penalver et al. [14]. However, the phytochemical content and nutrient profiles can exert significant variation, mainly ascribed to different locations, growth conditions, and seasonality, even within species [15]. Algae are generally considered a sustainable source of non-animal-derived proteins, rich in amino acids (especially glycine, alanine, arginine, proline, glutamic, and aspartic acids) essential for the human diet, whose content is often similar or higher to other traditional plant or animal sources [1,16]. Although algae show a low lipid content, they can have a high portion of unsaturated and polyunsaturated fatty acids, triglycerides, sterols, glycolipids, and phospholipids [1,14]. For this reason, algae are often categorized as a low-calorie food. Regarding their health benefits, it has been proven that regular consumption of seaweed can improve intestinal function and can promote antioxidant, anti-inflammatory, antimicrobial, antiviral, antihypertensive, antihyperlipidemic, immunomodulatory, anticancer, antidiabetic and anticoagulant activities, weight management, and disease prevention [8,13,17,18,19,20,21,22].

The quality evaluation of algae involves assessing their physicochemical and nutritional components to determine their suitability for specific end uses, which is generally performed using destructive, labor-intensive, expensive, and time-consuming traditional chemical analyses. In recent years, the hyperspectral imaging (HSI) technique has been successfully applied in food quality assessment and control [23]. It combines computer vision technology and spectroscopy to identify the sample’s two-dimensional images and one-dimensional spectral information [24]. Therefore, each image contains physical properties, such as shape, texture, and color, and spectral bands, which underline the chemical traits of the food product [24,25,26]. The main advantage of this technique is the ability to acquire a large amount of data from large-scale samples, obtaining chemical and physical information to qualitatively/quantitatively characterize complex food matrixes. Information from these large datasets can be managed to develop the calibration and validation of predictive models through appropriate chemometric tools. The rtificial neural network (ANN), one of the most common machine learning techniques, have gained attention due to their ability to reliably and practically predict food quality traits [27]. Compared to other linear algorithms, such as partial least square (PLS) regression, ANN can iteratively learn, identify, and model complex and often nonlinear relationships between the dependent and independent variables in the function of the provided patterns and without requiring prior knowledge of the relationships between variables [27,28]. 

In light of these considerations, our study aimed to evaluate the nutritional profile (proteins, lipids, and fibers) of seventeen macro- and microalgae species widely consumed around the world; to evaluate, for the first time, the predictive performance of a shortwave infrared (SWIR) hyperspectral imaging device (935–1720 nm), developing multi-species calibration models using ANN algorithms for three nutritional parameters (proteins, lipids, and fibers); to analyze prediction accuracy with different metrics; and to highlight the most predictive input spectral regions for each model. To the best of our knowledge, no studies have investigated the potential of shortwave infrared hyperspectral imaging devices to develop predictive models for the assessment of the nutritional parameters of algae.

## 2. Materials and Methods

### 2.1. Algae Material

Forty-one macro- and microalgae samples of different origins were considered in this study, as described in Table 1. The samples belonged to seventeen species widely consumed around the world, namely *Limnospira platensis*, *Auxenochlorella pyrenidosa*, *Chlorella vulgaris*, *Chondrus crispus*, *Eisenia bicyclis*, *Himanthalia elongata*, *Laminaria digitata*, *Laminaria longissima*, *Laminaria ochroleuca*, *Palmaria palmata*, *Porphyra umbilicalis*, *Pyropia yezoensis*, *Sargassum fusiforme*, *Ulva lactuca*, *Ulva lactuca* var. *spiralis*, *Ulva pertusa*, and *Undaria pinnatifida*. The samples were grown in different environments and were purchased from Italian markets over three years. The samples was ground by a Bühler MLI 203 sifter (Milan, Italy) and sieved to obtain a fine and homogeneous flour with particle sizes from 400 to 500 μm. Four aliquots were randomly extracted from each sample, on which the analyses were carried out.

### 2.2. Proximate Composition

Determination of the proximate composition of the algae was performed in triplicate and the data were expressed as g 100 g^−1^ on a dry weight basis (dw). The proteins and lipids were determined by the ICC standard methods 105/2 and 136, respectively [29]. Protein content was estimated using the conversion factor 5.0 instead of 6.25. In fact, recent studies highlighted that the use of a conversion factor equal to 6.25 overestimated the algae protein content and they identified the value 5.0 as the most suitable nitrogen-to-protein conversion factor [30,31,32,33]. Total dietary fiber content was measured according to Lee et al. [34].

### 2.3. Hyperspectral Imaging

**Figure 1:**
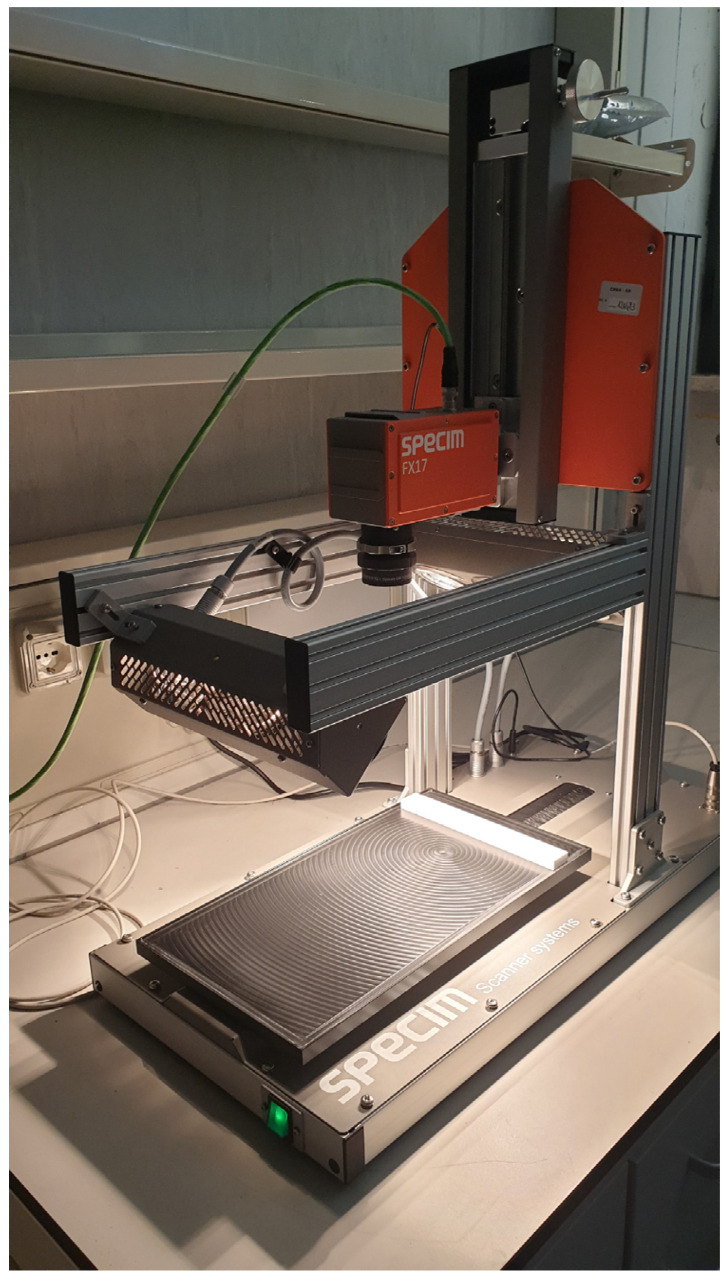
Shortwave infrared (SWIR) hyperspectral imaging device.

Shortwave infrared (SWIR) hyperspectral images of the algae samples (5 g of material in a Petri dish) were acquired in reflectance mode using a SisuCHEMA Hyperspectral Chemical Imaging Analyser (SPECIM, Spectral Imaging LTD, Oulu, Finland) system, as well described by Amoriello et al. [24]. The system (Figure 1) consists of a scanner table having a maximum scanning rate of 60 mm/s and a spatial resolution of 600 μm, with an integrated SPECIM diffusive line illumination unit, and a monochrome InGaAs image sensor detector (Specim FX17, Spectral Imaging Ltd., Oulu, Finland) with a spectral range of 935–1720 nm, a spectral resolution of 8 nm, and a spatial resolution of 640 pixels. The images were acquired and converted to spectral reflectance with Lumo-Scanner software (version 2022, Lumo-Scanner, Specim, Spectral Imaging Ltd., Oulu, Finland). The exposure time of the hyperspectral camera was set to 4.70 ms, the frame rate to 15.20 Hz, the positioning speed of the platform to 20.00 mm s^−1^, and the scanning speed to 5.84 mm s^−1^. The reflectance of the acquired hyperspectral images was calibrated using the white and dark reference images, according to the following equation:

$$R=\frac{R_{raw}-R_{B}}{R_{W}-R_{R}}$$

where R = the corrected reflectance, R_raw_ = the original reflectance, R_B_ = the black reference, and R_W_ = the white reference.

The HSI images were processed using Evince software (version 2.7.12, Prediktera AB, Umeå, Sweden). A principal component analysis (PCA) algorithm was used for the image segmentation and to remove the background, as described by Amoriello et al. [24]. Then, the reflectance spectra were smoothed with a baseline correction and the application of the first-order Savitzky–Golay filter for noise reduction. The light scattering was minimized using standard normal variate (SNV) correction. The mean spectrum was calculated as the average of the spectra related to all of the pixels of each sample, considering the overall hyperspectral image. All mean spectra were transformed by first-derivative treatment with a central difference approach to highlight species differences.

### 2.4. Artificial Neural Networks

**Figure 2:**
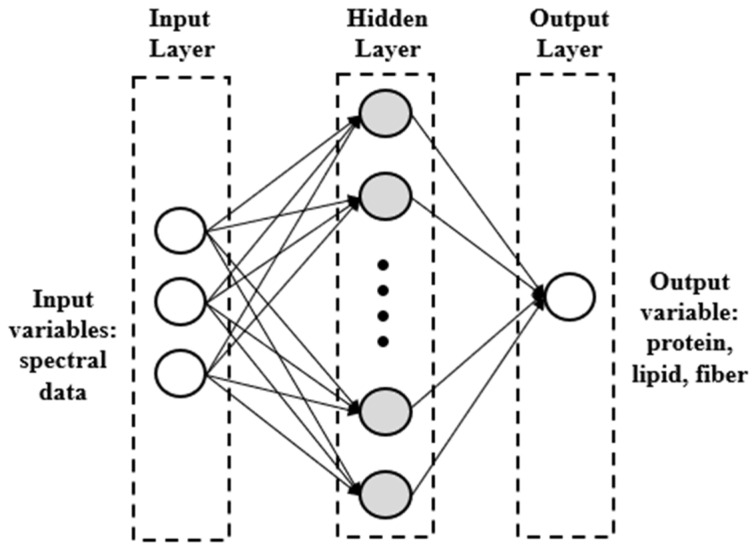
Structure of multilayer perceptron artificial neural network.

Three nutritional parameters (protein, lipid, and total fiber) were predicted using artificial neural network (ANN). The feed-forward architecture of the ANN, i.e., multilayer perceptron (MLP), combined with the Levenberg–Marquardt learning algorithm, was used to develop nonlinear models for the nutritional variables. The SWIR spectra of each algal sample represented the independent variables, and the dataset was randomly split into a training set (70% of the data), a testing set (15% of the data), and a validation set (15% of the data). The ANN architecture (Figure 2) was composed by three main layers: an input layer, which contains 164 spectral data, an output layer, i.e., the three nutritional parameters, and a hidden layer, as well described by Amoriello et al. [27]. 

Four activation functions (identity function, logistic function, hyperbolic tangent function, and exponential function) applied in the hidden or output layers and different topologies with different neurons in the hidden layer (from 1 to 25) were tested to evaluate the best topology for each model. The training process of the network was run 100,000 times with random initial values of weights and biases. The prediction performances were assessed using different metrics: the coefficient of correlation between observed and predicted values (r), the coefficient of determination (R^2^), the mean absolute error (MAE), the root mean squared error (RMSE), and the relative standard error (RSE), as described by Amoriello et al. [27]. The models and sensitivity analyses were developed using TIBCO^®^ Statistica statistical package software (version 13.5, TIBCO software Inc., Palo Alto, CA, USA).

### 2.5. Statistical Analysis

Differences between all of the nutritional variables were determined using the Kruskal–Wallis non-parametric test and Dunn’s post hoc test at a significance level of 5%, using PAST statistical software (version 4.17).

## 3. Results and Discussion

### 3.1. Exploratory Analysis

Table 2 summarizes the nutrient composition in terms of protein, lipid, and total fiber contents of different algae species. Protein content differed significantly between samples, showing the highest mean values for *Limnospira platensis* (62.1 ± 3.2 g 100 g^−1^ dw), formerly known as Spirulina, for *Auxenochlorella pyrenidosa* (59.0 ± 0.2 g 100 g^−1^ dw), and for *Chlorella vulgaris* (57.9 ± 0.2 g 100 g^−1^ dw). Conversely, the lowest values were recorded for brown algae, particularly by *Laminaria ochroleuca* (2.2 ± 0.2 g 100 g^−1^ dw, sample 23), in accordance with the results of Salido et al. [1] and Penalver et al. [13], which suggested a protein content near or below 15 g 100 g^−1^ dw for brown algae. The Food and Agriculture Organization of the United Nations (FAO) and the World Health Organization (WHO) recommend the consumption of Spirulina and Chlorella microalgae in the diet due to their high protein content, up to 70% protein per unit of dry weight [14]. Furthermore, these algae are composed of essential amino acids, suitable for human nutrition [5]. For these reasons, these microalgae are considered as a desirable and sustainable ingredient for protein supplements, to be consumed especially in vegetarian or vegan diets. However, seaweeds can also be considered a good source of protein due to their overall protein level and their amino acid composition [34]. For example, in our study, red algae also showed a good average protein content, from the 15.4 ± 1.4 g 100 g^−1^ dw of *Chondrus crispus* to the 26.6 ± 3.4 g 100 g^−1^ dw of *Porphyra umbilicalis*, as previously reported by Salido et al. [1] and Sultana et al. [35]. However, the differences in the chemical composition of algal species can be due to geographical location and environmental factors, such as seasonality, year, salinity, water temperature, and light irradiation, which could influence the nutrient supply, including the nitrogen availability [1,36].

The total lipid content of the algal species is quite low, ranging between the 0.6 ± 0.1 g 100 g^−1^ dw of *Pyropia yezoensis* and the 10.1 ± 0.1 g 100 g^−1^ dw of *Auxenochlorella pyrenidosa*. Generally, seaweeds contain limited lipid quantities (around 1–5%), whereas microalgae exhibit higher values (10–12%) [4,14,37,38]. Our study confirmed this lipid content. Slight discrepancies can be due to some physical factors, such as sunlight intensity, temperature, nutrient limitation, pH, and oxidative stress, which can affect lipid biosynthesis and composition, as described by Morales et al. [38] and Breuer et al. [39]. Algal lipid fraction is mainly composed of neutral lipids, such as fatty acids (especially, omega-3 polyunsaturated fatty acids), triglycerides and sterols, and complex lipids, such as glycolipids and phospholipids [40,41,42]. For this reason, algae are recognized for their health benefits and used in functional foods and nutraceuticals. 

Some edible algae are an important source of fiber, especially algae belonging to the Pheophyceae (brown algae) and Rhodophyceae (red algae) phylum. *Eisenia bicyclis* showed the highest fiber content (66.9 ± 0.3, 66.6 ± 0.3. 63.2 ± 0.3 g 100 g^−1^ dw for samples 12, 13, and 11, respectively), followed by *Sargassum fusiforme* (61.4 ± 0.3 g 100 g^−1^ dw), whilst *Limnospira platensis* showed the lowest (2.5 ± 0.2 g 100 g^−1^ dw, Sample 9). Similar fiber ranges for algae were reported by other authors, except for the *Limnospira platensis* values [4,14]. Algae are an excellent source of fibers, particularly soluble fibers (50–85% dw), such as alginates, fucoidans, carrageenans, and exopolysaccharides, contrary to the typical composition of fibers in terrestrial plants [13,43]. Due to their high fiber content, algae can contribute to a more balanced diet, enhancing daily fiber intake.

### 3.2. Spectral Characteristics

**Figure 3:**
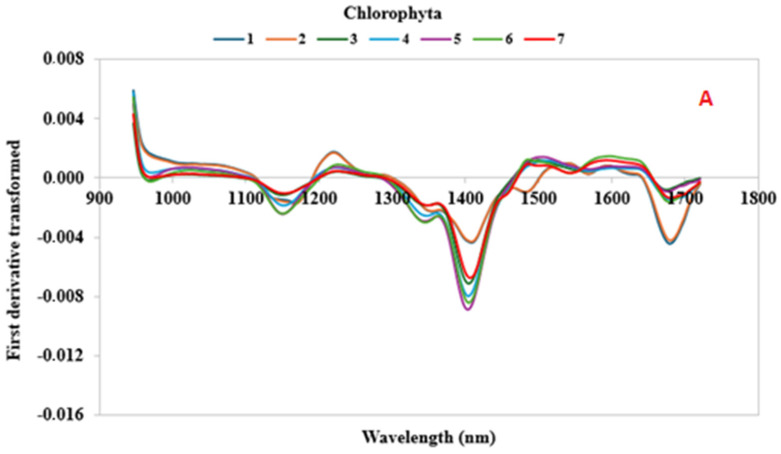

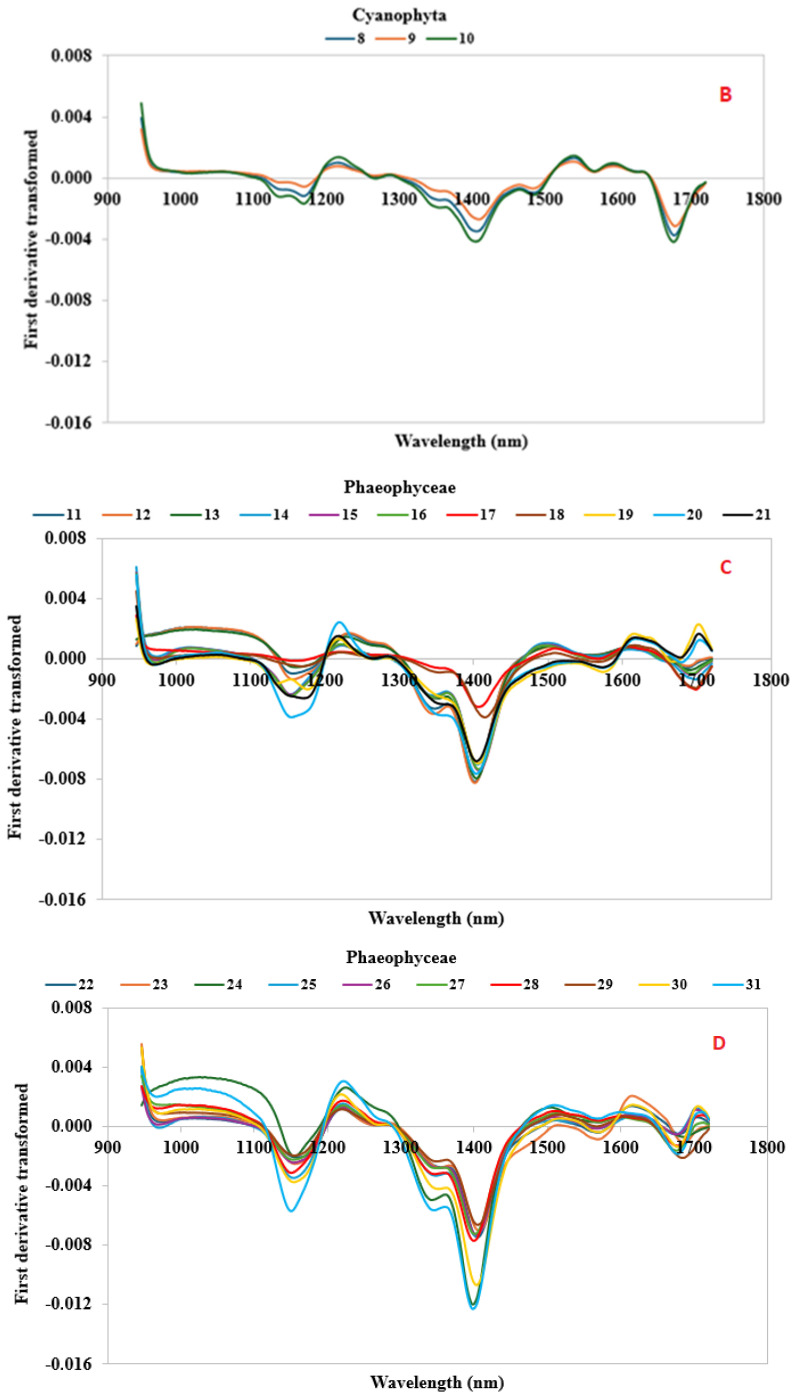

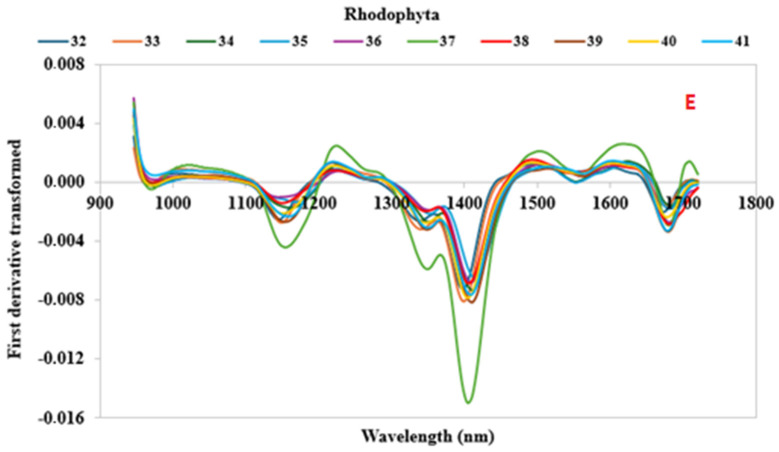
Mean first-derivative reflectance spectra between wavelengths of 935 and 1720 nm for 41 algae samples. Samples was clustered in relation to the phylum in five subfigures (**A**–**E**). The species of the samples identified by the numbers are reported in Table 1.

The SWIR first-derivatives spectra (935–1720 nm) were depicted in Figure 3 and contained information on different functional groups of the algae samples. The mean SWIR raw spectra of each algae sample are represented in Supplementary Figure S1. In general, the SWIR regions were mainly characterized by second-overtone spectral regions, which could be associated with the aliphatic chain (C–H_n_), hydroxyl group (O–H), and aminic group (N–H) characteristics of complex carbohydrates (cellulose, lignin, etc.), lipids, water, and proteins [44].

Functional groups, such as C–H, O–H, and N–H, were typical in the molecules of the biochemical substances of the algae. Qualitative and quantitative NIR analyses are often based on these [45]. The reflectance spectra showed similar profiles characterized by six notable peaks and of different magnitudes among all of the algae samples. The broad spectral region between 1100 and 1300 nm could be mainly referred to the C–H and C–H_2_ stretching vibration [46]. The prominent peak at around 1400 nm was assigned to O–H and N-H stretching of the first and second overtone. The signals in the wavelength region from 1600 nm to 1700 nm were caused by C–H and C–H_2_ vibrations [47].

In general, differences in the peaks’ intensities in the SWIR spectra between the different samples, especially in the regions between 1350 and 1450 nm, could be mainly related to the compounds, such as proteins, lipids, carbohydrates, and water contents, typical of the various algal species and phyla. At the same time, the geographical origin, the growth conditions, and the nutritional input could have influenced the formation and content of the chemical components and could have caused significant variability in the samples, as shown by the spectra [33,48,49].

Among the samples belonging to the Chlorophyta phylum (Figure 3A), Samples 2 and 4 (*Chlorella vulgaris* and *Ulva lactuca*) showed different spectral profiles in the regions between 1100 and 1200 nm, 1350 and 1450 nm, 1650 and 1700 nm, and prominent peaks at around 950 nm, 1200 nm, and 1500 nm. Conversely, Cyanophyta samples showed similar characteristic peaks, albeit with different signal intensities (Figure 3B).

The spectra of the Phaeophyceae samples (Figure 3C,D) were characterized by high variability in profile and intensity. The spectral signals for Samples 17 and 18 (*Laminaria digitata* from Northwest France and *Laminaria digitata* from the North Atlantic) were the lowest, whereas those of Samples 24, 25, and 30 (*Sargassum fusiforme*, *Undaria pinnatifida* from Japan, and *Undaria pinnatifida* from the Atlantic, respectively) the highest at between 900 and 1300 nm. Regarding to the Rhodophyta phylum samples, Sample 37 (*Palmaria palmata* from the Atlantic) showed a spectral profile very different from those of other red algae, especially for the regions between 1100 and 1200 nm, 1350 and 1450 nm, and 1650 and 1700 nm. Furthermore, prominent peaks at 950 nm, 1200 nm, and 1500 nm can be observed (Figure 3E).

These results demonstrate that the SWIR device can capture the intraspecies spectral differences, probably derived from the different growth conditions of the algae, and interspecies differences, in relation to the quantity of macroconstituents indicated by the band intensity at well-defined wavelengths.

### 3.3. ANN Model Prediction

**Figure 4:**
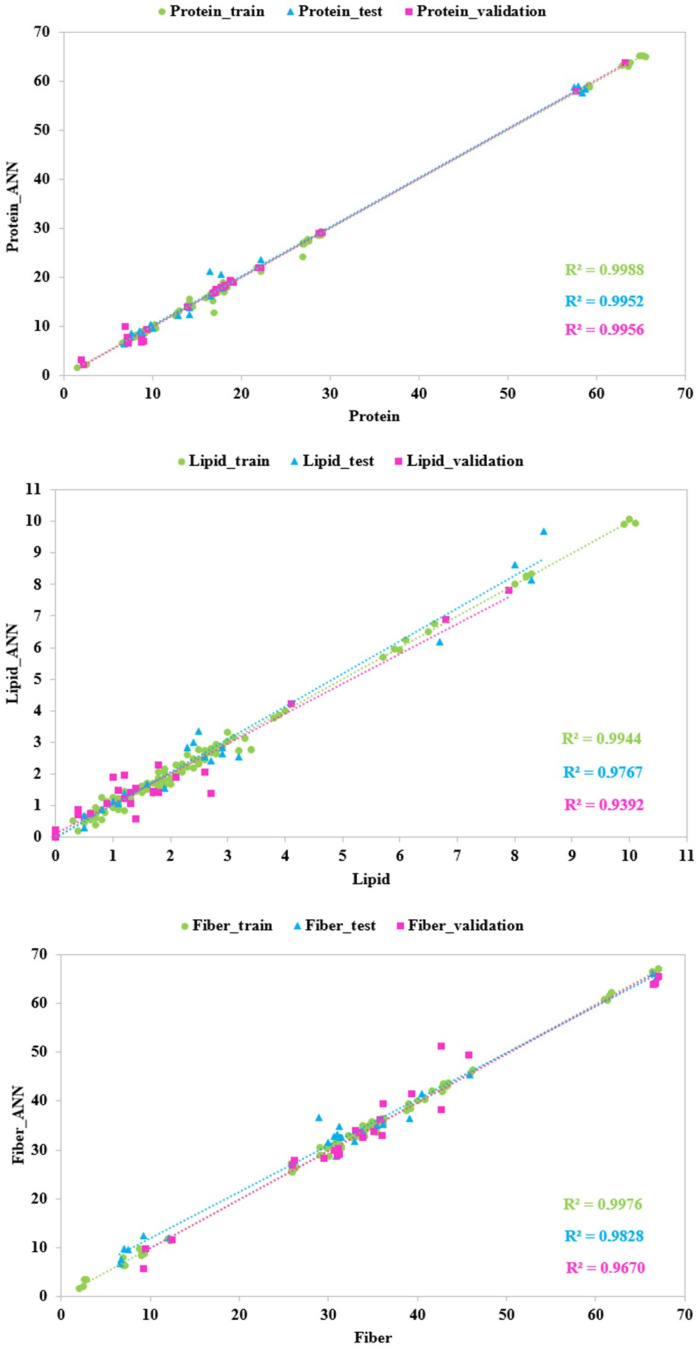
Predicted vs. experimental values of the protein, lipid, and fiber contents using the optimal ANN topologies and first-derivative transformed SWIR spectra. The coefficients of determination (R^2^) for the training, test, and validation sets are reported.

**Figure 5:**
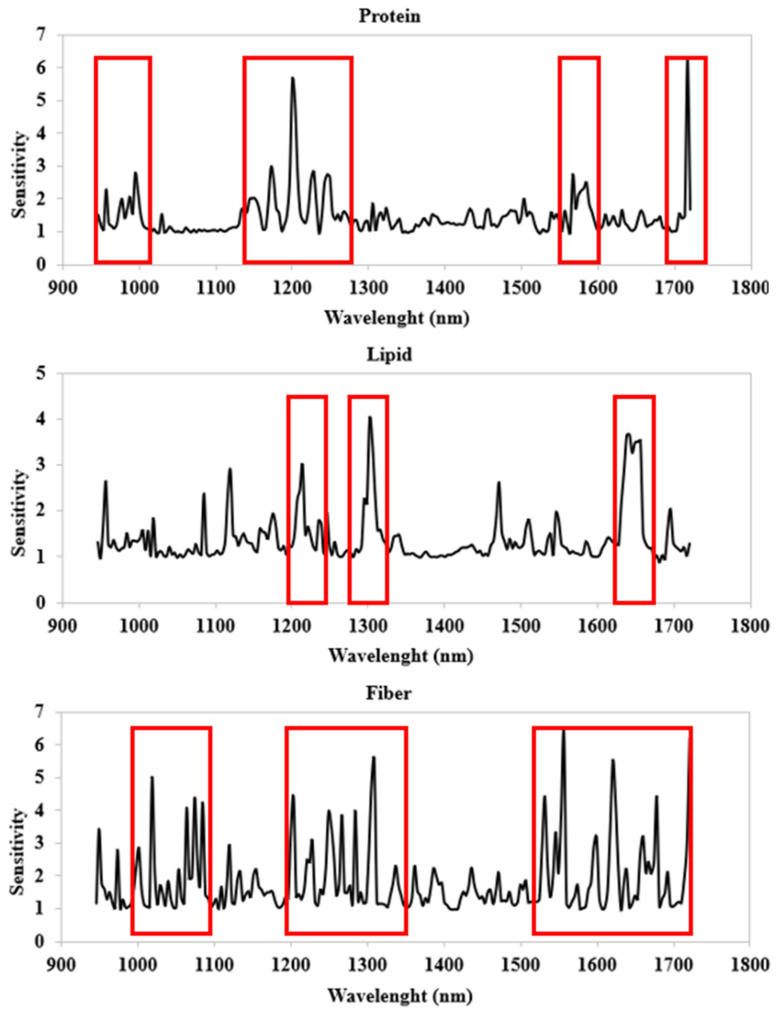
Results of the sensitivity analysis for each ANN model. The most sensitive spectral regions are highlighted in red.

The ANN models were developed using first-derivative transformed spectral data. The ANN activation functions for the best topology generated for each output variable and the modeling performance in terms of the coefficient of correlation (r), the coefficient of determination (R^2^), the mean absolute error (MAE), the root mean squared error (RMSE), and the relative standard error (RSE) for the training, test, and validation sets are shown in Table 3 and Figure 4, whilst the results from the sensitivity analysis for each ANN model are reported in Figure 5. The best five ANN architectures for each parameter are shown in Table S1 (Supplementary Materials).

Sensitivity analysis is one of the most widely used methods to rate the importance of the models’ input variables. It is based on the partial derivatives method, and it consists of calculating the derivative of the output regarding each input variable of the neural network, evaluated on each data sample of a given dataset, as described by Pizarroso et al. [50]. The contribution of each input is calculated in both magnitude and sign considering the connection weights, the activation functions, and the values of each input. Once the sensitivity has been calculated for each variable and observation, a global sensitivity can be defined considering the sum of the derivatives of the output of the k-th neuron in the output layer regarding the i-th input variable divided by the number of samples. If variable is important, the global sensitivity should be large (>>1).

All models showed optimal prediction accuracy. In detail, the best model for the protein content was obtained with 11 neurons in the hidden layer, and a hyperbolic tangent activation function for the hidden and output neurons. The very high values of r and R^2^ (0.9976 and 0.9952, respectively) and the low values of RMSE, MAE, and RSE (1.2891, 0.2590, and 5.8128, respectively) for the test set showed an excellent prediction performance. Sensitivity analysis (Figure 5) showed bands with high intensity peaks in the spectral range at around 1700–1750 nm and at around 1175–1225 nm, followed by several bands between 950 and 1000 nm, 1125 and 1175 nm, 1250 and 1300 nm, and 1560 and 1600 nm, characterized by lower intensities. Indeed, the spectral regions close to 1730 nm and 1200 nm were characterized by S–H first- and second-overtone absorption, whereas in the other regions a N–H first and second overtone was observed. Niemi et al. [33] reported a similar spectral range contribution to the development of a FT-NIR prediction model of protein in North Atlantic seaweeds. Specifically, they found positive correlation between the major protein bands at around 1050–1350 nm, 1550–1600 nm, and 1700–1750 [33].

The best model for the lipid content, built with 24 neurons in the hidden layer, an exponential function for the hidden neurons, and a logistic activation function for the output neurons, presented an optimal predictive ability due to values of r and R^2^ equal to 0.9823 and 0.9767, respectively, and due to high values of the other metrics for the test set (RMSE = 0.4096 and MAE = 0.0834). Although the RSE metric is quite high (RSE = 15.6521), the estimate may still be considered reliable because the value does not exceed the 30% threshold, as indicated by Amoriello et al. [27]. Sensitivity analysis (Figure 3) revealed that the bands of lipids with high intensity peaks were centered at around 1195–1215 nm for the C–H_3_ and C–H_2_ second overtone of C–H stretch and at around 1290–1310 nm for the C–H_3_ first overtone of C-H stretch. Absorptions at around 1680 nm were contributed by C–H stretch (–CH=CH–) and can be used to quantify unsaturated fatty acids [48,49]. As previously reported by Liu et al. [51], the NIR spectra within the wavelength ranges of 1030–1500 and 1600–1880 nm correspond to the area where fatty acids show dominant absorbance.

A high predictive accuracy was also found for best model of fiber content, developed with 10 neurons in the hidden layer, an exponential function for the hidden neurons, and an identity function for the output neurons. In fact, metrics for the test set were 0.9914, 0.9828, 2.3032, 0.7968, and 7.5954 for r, R^2^, RMSE, MAE, and RSE, respectively. Sensitivity analyses highlighted the wavebands that exhibited the best predictive ability for fiber content (Figure 5). The spectra regions that contribution to the model development can be attributed to O-H bending and C–H stretching (characteristic of wave lengths between 1000 nm and 1100 nm), C–H stretching (characteristic of spectral bands between 1200 nm and 1350 nm), C–H_3_, C–H_2_, and C–H stretching (characteristic of wave bands between 1550 nm and 1720 nm), which mainly form carbohydrates [52,53].

The good performances of the ANN models for the three algal nutritional parameters highlighted the advantages of the use of the ANN technique to develop accurate prediction models. Ordinary statistical techniques, such as partial least square (PLS) regression, are not always able to precisely quantify the complex inter- and intra-relations between input and output variables [54]. Conversely, ANN, inspired by the biological neural network comprising the human brain, has numerous advantages. First of all, it has the ability to solve complex nonlinear relationships between dependent and independent variables, learning iteratively the characteristics of algae via the extraction of features from a large database (i.e., the spectral data). No prior knowledge of the relationships between the process variables, no constraints on input variables, and no fixed relationships in the data are required [55]. Then, the ANN can also be used for unstable, noisy, imprecise, and incomplete data [56].

The high predictive accuracy of the models can also be due to having considered many algal genotypes, thus obtaining multi-species models. In fact, according to Gholipoor and Nadali [54], the models could be more reliable the greater the number of genotypes, and therefore the greater the variability, if the genotypes are substantially different in terms of traits.

Finally, the sensitivity analysis made it possible to highlight the most important wavelengths of the three models. This result can be useful to build less expensive devices to use in screening or process control applications in the algal industry.

## 4. Conclusions

The nutritional profile of macro- and microalgae species can vary a great deal and their biochemical properties are strongly influenced by algal genotype, growth and environmental conditions, and nutrient availability. A fast, easy, and non-destructive assessment of the qualitative and quantitative characteristics of algae is increasingly requested by food industry. The overall results demonstrated that the use of an SWIR hyperspectral imaging device combined with machine learning techniques was able to successfully predict the algal composition. In fact, the multi-species models for proteins, lipids, and fibers, developed with the artificial neural network, showed excellent and robust predictive performances. A goal of this study was a reduction in species-specific influences using the SWIR spectra of seventeen algal species of different phylogenetic divisions. Therefore, the developed models can be applied to any species with a high confidence level. Moreover, the sensitivity analysis enabled the identification of the most informative spectral wavelengths and those that were redundant and irrelevant. Although our results are very promising, a further test of the goodness of the models needs to be conducted on unknown samples before its application on a routine basis in food industries.

## Figures and Tables

**Table 1:** Samples of algae divided by classes, species, and origin.

Sample Number	Phylum	Species	Origin
1	Chlorophyta (green algae)	*Auxenochlorella pyrenidosa*	Northwest France
2		*Chlorella vulgaris*	China
3		*Ulva lactuca*	Northwest Spain
4		*Ulva lactuca*	Northwest France
5		*Ulva lactuca*	Italy
6		*Ulva lactuca* var. *spiralis*	Ireland
7		*Ulva pertusa*	Japan
8	Cyanophyta (blue-green algae)	*Limnospira platensis*	Italy
9		*Limnospira platensis*	China
10		*Limnospira platensis*	Italy
11	Phaeophyceae (brown algae)	*Eisenia bicyclis*	Japan
12		*Eisenia bicyclis*	Japan
13		*Eisenia bicyclis*	Japan
14		*Himanthalia elongata*	Northwest Spain
15		*Himanthalia elongata*	Northwest France
16		*Himanthalia elongata*	Northwest France
17		*Laminaria digitata*	Northwest France
18		*Laminaria digitata*	North Atlantic
19		*Laminaria digitata*	Japan
20		*Laminaria longissima*	Japan
21		*Laminaria longissima*	Japan
22		*Laminaria ochroleuca*	Atlantic
23		*Laminaria ochroleuca*	Atlantic
24		*Sargassum fusiforme*	Atlantic
25		*Undaria pinnatifida*	Japan
26		*Undaria pinnatifida*	Northwest Spain
27		*Undaria pinnatifida*	Atlantic
28		*Undaria pinnatifida*	Korea
29		*Undaria pinnatifida*	Northwest Spain
30		*Undaria pinnatifida*	Atlantic
31		*Undaria pinnatifida*	Northwest Pacific
32	Rhodophyta (red algae)	*Chondrus crispus*	Ireland
33		*Chondrus crispus*	Ireland
34		*Palmaria palmata*	Atlantic
35		*Palmaria palmata*	Atlantic
36		*Palmaria palmata*	Ireland
37		*Palmaria palmata*	Atlantic
38		*Porphyra umbilicalis*	Southwest Atlantic
39		*Porphyra umbilicalis*	Argentina
40		*Porphyra umbilicalis*	Atlantic
41		*Pyropia yezoensis*	Northwest France

**Table 2:** Nutrient composition (protein, lipid, and total fiber) of algal samples.

Sample Number	Species	Protein (g 100 g^−1^ dw)	Lipid (g 100 g^−1^ dw)	Fiber (g 100 g^−1^ dw)
1	*Auxenochlorella pyrenidosa*	59.0 ± 0.2 c	10.1 ± 0.1 a	9.0 ± 0.2 r
2	*Chlorella vulgaris*	57.9 ± 0.2 d	6.7 ± 0.1 c	12.2 ± 0.2 q
3	*Ulva lactuca*	16.9 ± 0.2 l	0.6 ± 0.1 m	34.6 ± 0.6 i
4	*Ulva lactuca*	16.5 ± 0.1 l	1.2 ± 0.7 il	34.6 ± 0.6 i
5	*Ulva lactuca*	16.9 ± 0.3 l	0.7 ± 0.1 m	35.0 ± 0.3 i
6	*Ulva lactuca* var. *spiralis*	12.8 ± 0.2 n	0.9 ± 0.1 l	40.7 ± 0.7 f
7	*Ulva pertusa*	27.4 ± 0.3 f	1.5 ± 0.2 i	43.1 ± 0.3 e
8	*Limnospira platensis*	63.3 ± 0.4 b	8.1 ± 0.2 b	6.9 ± 0.3 s
9	*Limnospira platensis*	65.1 ± 0.3 b	5.9 ± 0.2 d	2.5 ± 0.3 t
10	*Limnospira platensis*	58.0 ± 0.4 d	8.3 ± 0.2 b	7.1 ± 0.3 s
11	*Eisenia bicyclis*	9.1 ± 0.3 op	1.6 ± 0.1 i	63.2 ± 0.2 b
12	*Eisenia bicyclis*	7.0 ± 0.2 r	1.2 ± 0.1 l	66.9 ± 0.3 a
13	*Eisenia bicyclis*	7.0 ± 0.3 r	1.1 ± 0.2 l	66.6 ± 0.3 a
14	*Himanthalia elongata*	7.8 ± 0.3 q	1.0 ± 0.1 l	35.9 ± 0.3 h
15	*Himanthalia elongata*	10.2 ± 0.1 o	2.6 ± 0.1 g	31.2 ± 0.3 n
16	*Himanthalia elongata*	10.0 ± 0.2 o	2.6 ± 0.2 g	31.1 ± 0.2 n
17	*Laminaria digitata*	8.8 ± 0.2 p	1.8 ± 0.1 i	35.9 ± 0.3 h
18	*Laminaria digitata*	9.2 ± 0.3 o	1.1 ± 0.1 l	33.4 ± 0.3 m
19	*Laminaria digitata*	8.8 ± 0.2 p	1.8 ± 0.1 i	36.1 ± 0.1 h
20	*Laminaria longissima*	7.9 ± 0.3 q	3.1 ± 0.1 f	32.6 ± 0.3 m
21	*Laminaria longissima*	8.8 ± 0.1 p	1.7 ± 0.2 i	36.0 ± 0.1 h
22	*Laminaria ochroleuca*	7.2 ± 0.1 r	2.1 ± 0.1 h	35.1 ± 0.3 i
23	*Laminaria ochroleuca*	2.2 ± 0.2 s	0.0 ± 0.0 n	9.4 ± 0.1 r
24	*Sargassum fusiforme*	6.8 ± 0.2 r	1.2 ± 0.1 l	61.4 ± 0.3 c
25	*Undaria pinnatifida*	17.5 ± 0.2 i	3.2 ± 0.2 f	31.0 ± 0.1 n
26	*Undaria pinnatifida*	16.9 ± 0.1 l	2.2 ± 0.1 h	33.0 ± 0.1 m
27	*Undaria pinnatifida*	16.9 ± 0.2 l	2.7 ± 0.2 g	31.1 ± 0.2 n
28	*Undaria pinnatifida*	14.2 ± 0.2 m	1.7 ± 0.1 i	35.1 ± 0.1 i
29	*Undaria pinnatifida*	17.1 ± 0.3 l	2.0 ± 0.3 h	34.0 ± 0.2 l
30	*Undaria pinnatifida*	16.8 ± 0.2 l	2.6 ± 0.1 g	30.8 ± 0.3 n
31	*Undaria pinnatifida*	17.9 ± 0.2 h	4.0 ± 0.1 e	29.2 ± 0.2 o
32	*Chondrus crispus*	16.7 ± 0.1 l	2.3 ± 0.1 h	30.6 ± 0.1 n
33	*Chondrus crispus*	14.1 ± 0.1 m	0.0 ± 0.0 n	46.0 ± 0.2 d
34	*Palmaria palmata*	18.2 ± 0.1 h	0.5 ± 0.1 m	31.1 ± 0.3 n
35	*Palmaria palmata*	27.0 ± 0.1 f	1.2 ± 0.1 l	43.0 ± 0.2 e
36	*Palmaria palmata*	17.9 ± 0.1 h	0.6 ± 0.1 m	31.0 ± 0.2 n
37	*Palmaria palmata*	27.2 ± 0.2 f	1.2 ± 0.2 l	42.9 ± 0.1 e
38	*Porphyra umbilicalis*	22.1 ± 0.1 g	0.0 ± 0.0 n	36.6 ± 0.1 h
39	*Porphyra umbilicalis*	29.0 ± 0.1 e	2.7 ± 0.2 g	26.2 ± 0.1 p
40	*Porphyra umbilicalis*	28.8 ± 0.2 e	2.6 ± 0.2 g	26.0 ± 0.2 p
41	*Pyropia yezoensis*	22.0 ± 0.2 g	0.6 ± 0.1 m	39.1 ± 0.2 g

Differences between letters (a–t) in the same column indicate significant differences (*p* < 0.05).

**Table 3:** Neural network architectures; regression metrics for the highest training, test, and validation sets predictions; goodness of fit; and residual analysis for the developed ANN models.

		Protein (g 100 g^−1^ dw)	Lipid (g 100 g^−1^ dw)	Fiber (g 100 g^−1^ dw)
**Activation Function**	Hidden Neurons	Tahn	Exp	Exp
	Output Neurons	Tahn	Logistic	Identity
**Training Set**	r	0.9994	0.9972	0.9988
	R^2^	0.9988	0.9944	0.9976
	MAE	0.0396	0.0027	0.0473
	RMSE	0.5593	0.1606	0.6350
	RSE	2.7248	7.0000	1.9405
**Test Set**	r	0.9976	0.9883	0.9914
	R^2^	0.9952	0.9767	0.9828
	MAE	0.2590	0.0834	0.7968
	RMSE	1.2891	0.4096	2.3032
	RSE	5.8128	15.6521	7.5954
**Validation Set**	r	0.9978	0.9691	0.9833
	R^2^	0.9956	0.9392	0.9670
	MAE	0.0250	0.0146	0.2701
	RMSE	0.9340	0.4534	2.7609
	RSE	5.0772	24.9689	7.7873

Tanh = hyperbolic tangent function; Exp = exponential function.

## Data Availability

The original contributions presented in the study are included in the article, further inquiries can be directed to the corresponding author.

## Supplementary Materials

The following supporting information can be downloaded at: https://www.mdpi.com/article/10.3390/foods13142277/s1, Figure S1: Mean raw reflectance spectra between 935 and 1720 nm wavelength of each algae sample; Table S1: Neural network architectures and correlation coefficients for the developed ANN models.
