# Supplementary material for: Evaluation of Nutritional Values of Edible Algal Species Using a Shortwave Infrared Hyperspectral Imaging and Machine Learning Technique
Source: Foods. 2024 Jul 19;13(14):2277. doi: 10.3390/foods13142277 (PMC11275431; doi:10.3390/foods13142277)
Supplement: Supplementary file 1 [file foods-13-02277-s001.zip › foods-3088160-supplementary.pdf]

Supplementary Materials

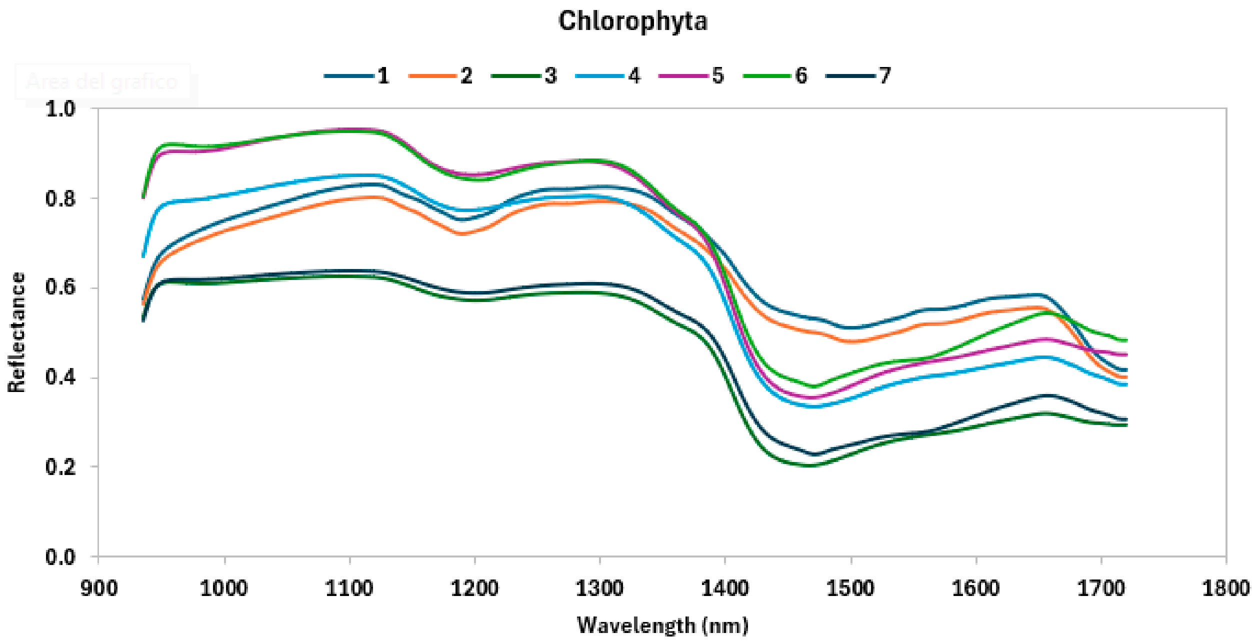

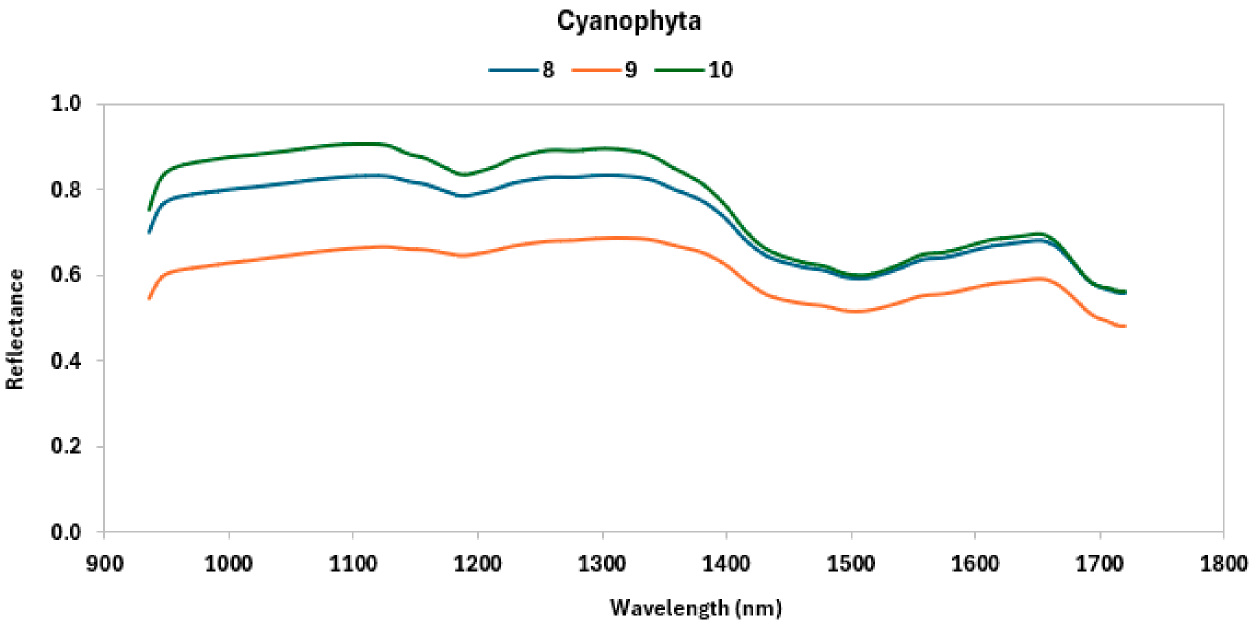

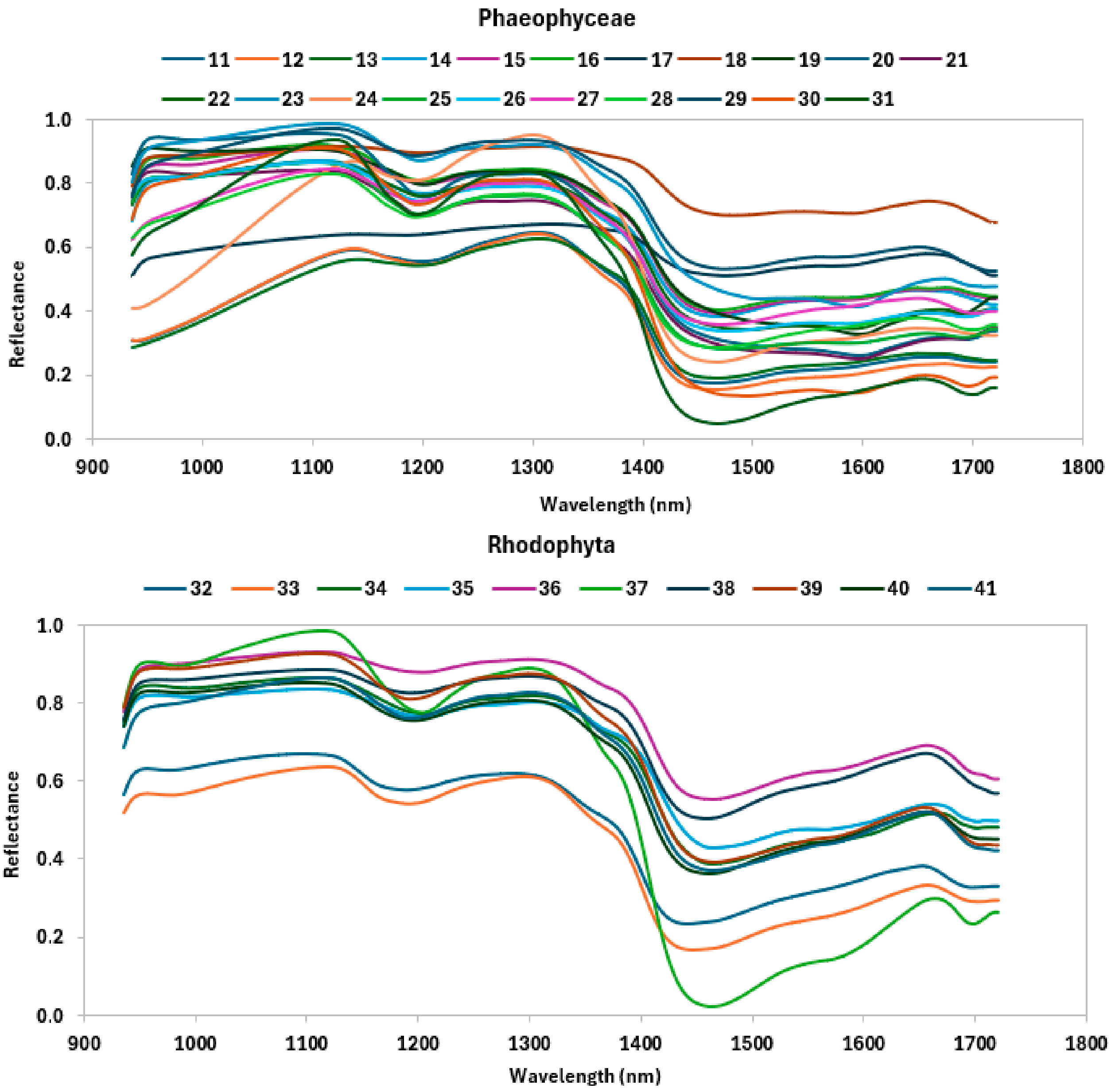

Figure S1. Mean raw reflectance spectra between 935 and 1720 nm wavelength of each algae sample.

Table S1. Neural network architectures and correlation coefficients for the developed ANN models.

	Neurons in hidden layers	Activation Function		Training Set	Test Set	Validation set
		**Hidden Neurons**	**Output Neurons**	**Correlation coefficient**	**Correlation coefficient**	**Correlation coefficient**
Protein	11	Tanh	Logistic	0.9979	0.9926	0.9929
	18	Tanh	Identity	0.9982	0.9953	0.9949
	9	Logistic	Logistic	0.9928	0.9938	0.9935
	**11**	**Tanh**	**Tanh**	**0.9994**	**0.9976**	**0.9978**
	12	Tanh	Logistic	0.9940	0.9953	0.9938
Lipid	**24**	**Exp**	**Logistic**	**0.9972**	**0.9883**	**0.9691**
	21	Exp	Identity	0.9911	0.9608	0.9602
	16	Tanh	Logistic	0.9869	0.9540	0.9485
	20	Tanh	Tanh	0.9629	0.9699	0.9603
	20	Tanh	Tanh	0.9730	0.9672	0.9749
Fiber	19	Exp	Tanh	0.9975	0.9871	0.9805
	18	Logistic	Tanh	0.9993	0.9861	0.9844
	**10**	**Exp**	**Identity**	**0.9988**	**0.9914**	**0.9833**
	22	Tanh	Logistic	0.9975	0.9888	0.9842
	22	Tanh	Logistic	0.9967	0.9887	0.9823

The best architecture for each parameter was highlighted in bold.
